# Potent Neutralization of Influenza A Virus by a Single-Domain Antibody Blocking M2 Ion Channel Protein

**DOI:** 10.1371/journal.pone.0028309

**Published:** 2011-12-02

**Authors:** Guowei Wei, Weixu Meng, Haijiang Guo, Weiqi Pan, Jinsong Liu, Tao Peng, Ling Chen, Chang-You Chen

**Affiliations:** 1 State Key Laboratory of Respiratory Disease, Guangzhou Institute of Biomedicine and Health, Chinese Academy of Sciences, Guangzhou, China; 2 State Key Laboratory of Respiratory Disease, Guangzhou Institute of Respiratory Disease, Guangzhou Medical University, Guangzhou, China; Centers for Disease Control and Prevention, United States of America

## Abstract

Influenza A virus poses serious health threat to humans. Neutralizing antibodies against the highly conserved M2 ion channel is thought to offer broad protection against influenza A viruses. Here, we screened synthetic Camel single-domain antibody (VHH) libraries against native M2 ion channel protein. One of the isolated VHHs, M2-7A, specifically bound to M2-expressed cell membrane as well as influenza A virion, inhibited replication of both amantadine-sensitive and resistant influenza A viruses *in vitro*, and protected mice from a lethal influenza virus challenge. Moreover, M2-7A showed blocking activity for proton influx through M2 ion channel. These pieces of evidence collectively demonstrate for the first time that a neutralizing antibody against M2 with broad specificity is achievable, and M2-7A may have potential for cross protection against a number of variants and subtypes of influenza A viruses.

## Introduction

As a serious public health threat, influenza A virus causes seasonal epidemics as well as occasional pandemics. It is estimated that 250,000–500,000 people die from influenza each year throughout the world [1]. The 1918 Spanish influenza pandemic infected close to 5% of the world's population and caused a devastating effect [2]. Recent outbreaks of H1N1 influenza (Swine flu) again raised serious concerns about potential influenza pandemics [3]. Although vaccines and anti-viral drugs are currently available to control influenza, their prophylactic and therapeutic effects remain incomplete. Conventional vaccines mainly target two highly variable determinants; namely, hemagglutinin (HA) and neuraminidase (NA). Due to rapid genetic drift and re-assortment of the viral genome, viral strains evolve continuously and necessitate frequent updates for vaccine production. The time delay from monitoring the emergences of new viral strains to producing effective vaccines at an industrial scale limits our ability to provide immediate protection when a pandemic occurs [4]. In turn, the new vaccines would not be able to provide effective protection for immuno-compromised individuals, young children and the elderly [5]. Besides vaccines, anti-viral drugs such as NA inhibitors zanamivir and oseltamivir as well as matrix-2 protein (M2) inhibitors amantadine and its derivative rimantadine were approved to combat influenza. However, substantial amount of drug-resistant viruses emerged due to frequent use of these drugs. Alarmingly, in humans, birds, and pigs, amantadine-resistant viruses constitute more than 90% of total [6]–[8]. Thus, there is a pressing need to develop effective prophylactic and therapeutic agents against infection of different variants and subtypes of influenza A viruses.

Influenza M2 is an integral tetrameric transmembrane protein that functions as a proton channel required for uncoating the virus in endosomes upon infection, and hence, a functional M2 is essential for a productive infection to occur [9]–[12]. Compared to other viral surface proteins such as HA and NA, the 23-amino acid extracellular domain of M2 (M2e) is remarkably conserved in all human influenza A viruses [13]. This distinctive characteristic makes M2e an attractive target for developing a “universal” vaccine. In recent years, several M2e-based vaccines have been demonstrated in animal models to protect against human and avian influenza infections [14]–[18]. However, inadequate antibody titers are particular challenging due to the low immunogenicity of M2e [19], and multiple injections of high-dose immunogens with an adjuvant are required to achieve high levels of neutralizing antibodies [20]. Passive immunization has been proven to be an effective and safe strategy for the prevention and treatment of viral diseases [21]. Passive transfer of murine anti-M2e antibody 14C2 significantly inhibited influenza A virus replication in mice [22]. Several groups developed M2e monoclonal antibodies (mAbs) and demonstrated their prophylactic and therapeutic activities against influenza [20], [23]–[26]. In general, these antibodies mediate protection by eliminating infected cells through antibody-dependent cell-mediated cytotoxicity (ADCC) or complement dependent cytotoxicity (CDC), not through neutralizing virions as M2 ion channel blockers [20], [27]. Conceivably, blocking M2 ion channel would be an effective antiviral approach since M2 is involved in virus uncoating at an early stage of the viral life cycle. Yet, there is no report about the approach designed to generate antibodies against M2 ion channel function.

Naturally occurring heavy-chain antibodies devoid of light chains were first discovered in *Camelidae* [28]. The antigen-binding site of these antibodies consists of one single domain referred to as VHH. With a molecular weight of approximately 15 kD, VHHs are the smallest naturally occurring intact antigen-binding units with the following noticeable features: (1) long CDR3 that plays a key role in recognizing structures such as pockets and clefts that are inaccessible for conventional antibodies [29]; (2) efficiently produced in prokaryotic and eukaryotic hosts including bacteria and yeast [30]; (3) generally not immunogenic in primates and can be humanized if necessary [31]; (4) highly soluble and stable including resistance to high temperature and proteases [32], [33]. A number of VHHs had been developed for a spectrum of human diseases, and some of which are currently in late stages of clinical trials. In this study, we explored the possibility of generating VHH antibodies specifically targeting native M2 ion channel. By panning and subtractive selection of synthetic Camel VHH libraries on native MDCK cells vs virus infected cells, a number of anti-M2 VHH antibodies were isolated. Among the VHHs, M2-7A, showed cross-reactive neutralization for both amantadine-sensitive and resistant viruses *in vitro* and protection from influenza A virus infection in mice. Using a cell viability assay, M2-7A was demonstrated to protect M2-expressing cells from pH shock-induced cell mortality. Our results suggest M2-7A may neutralize M2 by interfering with its ion channel function and have the potential to become cross protective anti-influenza agents.

## Materials and Methods

### Expression and purification of the full length M2 protein

The M2 gene of an influenza virus (A/Hong Kong/8/68, H3N2) was cloned into pET32a(+) vector (Novagen) and expressed in *E. coli* BL21(DE3) (Novagen). The expressed His-tagged protein was first purified by immobilized metal-ion affinity chromatography (IMAC). The Trx tag was then cleaved by thrombin and further purified by AKTA (GE Healthcare) through ion exchange chromatography using Source Q column (GE Healthcare), HisTrap FF affinity chromatography (GE Healthcare), and gel filtration using Superdex 200 column (GE Healthcare). Detergent (1% OG) was included in all purification buffers. Protein purity was detected by Coomassie-stained sodium dodecyl sulfate-polyacrylamide gel electrophoresis (SDS-PAGE) and the concentration was determined by a protein assay kit (Bio-Rad).

### Oligonucleotide design for PCR-based gene synthesis

An antibody library was constructed based on the identified universal VHH framework cAbBCII10 with synthetic diversity introduced by PCR mutagenesis into all three complementarity determining regions (CDR1-3) [34]. DNA degeneracy is represented by the IUB code (D = A/G/T, K = G/T, M = A/C, N = A/C/G/T, R = A/G, S = G/C, V = A/C/G, W = A/T, Y = C/T). Degenerate codons are shown in bold text. Mutagenic oligonucleotides used for library constructions are: Forward primer, GTC CTC GCA ACT GCG GCC CAG CCG GCC ATG CAG GTG CAG CTG GTA GAA TCA GGC GG; Oligo1(FR1), CAG GTG CAG CTG GTA GAA TCA GGC GGT GGC **TYG** GTA CAG GCC GAA GGT TCG TTG CGT TTG TCC TGT **RCT** GCC TCG GGT ; Oligo2(CDR1-FR2), GGC GGC AAC **MAA** TTC ACG TTC TTT ACC TGG AGC CTG GCG **GWA** CCA ACC **MAT ASM AYW ANT RCT VRA RRT ADA** ACC CGA GGC **AGY** ACA GGA CAA; Oligo3(CDR2-FR3), ATC ACG TGA AAT AGT AAA ACG GCC CTT GAC GGA GTC TGC ATA **GTN TGT KBT GBC AYC AYY CVW ABT** AAT GGC GGC AAC **MAA** TTC ACG TTC; Oligo4(FR3), CGT TTT ACT ATT TCA CGT GAT AAT GCC AAA AAT ACT GTC TAT TTG CAG ATG AAT **ART** TTG AAA CCA GAA GAT ACT GCC RTT TAT TAC TGT; Oligo5(CDR3-FR4), TGA TGA GAC AAT GAC **MTG** GGT CCC TTG GCC CCA GTA **MNN** **(6–17)** GGC **AKY** ACA GTA ATA **AAY** GGC AGT AT; Reverse primer, GAG TCA TTC TCG ACT TGC GGC CGC TGA TGA GAC AAT GAC MTG GGT CCC.

### VHH library construction

**Figure 1:**
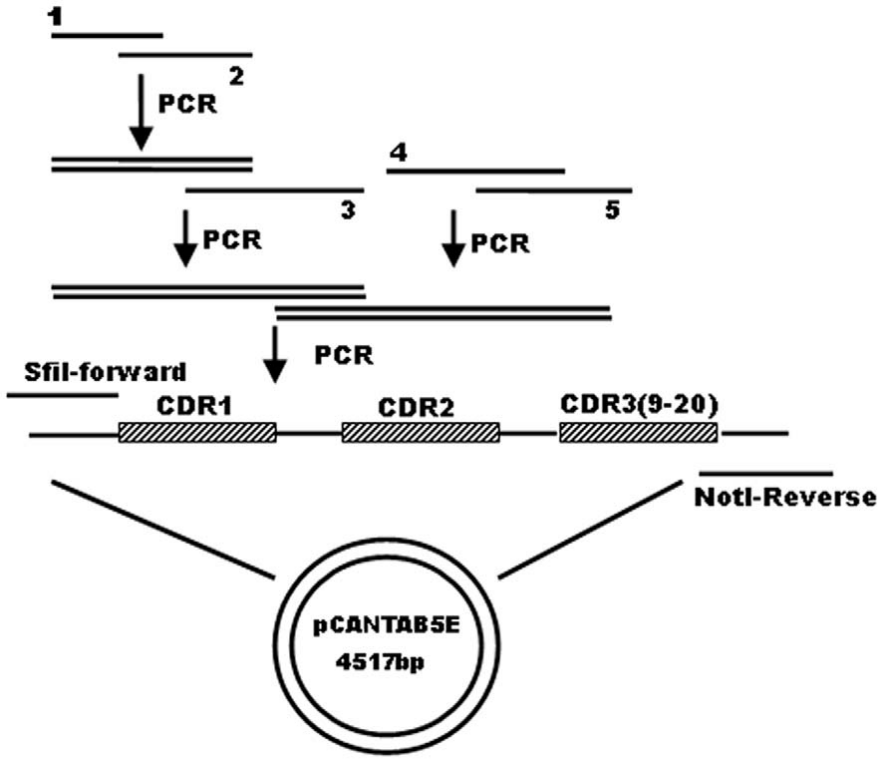
Schematic flow chart for the assembly of the synthetic oligonucleotides. Synthetic VHH genes were generated by PCR extension of oligonucleotides represented by oligo1-5 (FR1, CDR1-FR2, CDR2-FR3, FR3, CDR3-FR4), forward and reverse primers. The final PCR products were purified from agarose gels, digested sequentially with *Sfi*I and *Not*I, and cloned into the pCANTAB 5 E phagmid vector.

Library construction was done according to the provider's instructions with minor changes (GE Healthcare, previously Amersham Biosciences). Synthetic oligonucleotides were assembled and amplified by overlap PCR extension, as illustrated in Fig. 1. The purified final PCR products and pCANTAB 5E phagemid vector (GE Healthcare) were digested with *Not*I and *Sfi*I (New England Biolabs), and subsequently gel-purified using a QIAquick Gel Extraction Kit (Qiagen). The resulting VHH fragments (∼5 µg) were ligated into pCANTAB 5E (∼10 µg) with T4 DNA ligase (New England Biolab) at 16°C for 16 h. The ligated material was transformed into competent TG1 cells (GE Healthcare) by several electroporations using a Bio-Rad Gene Pulser (Bio-Rad Laboratories). Each library was grown at 30°C overnight on plates containing 2×YT medium, supplemented with ampicillin (100 µg/mL) and glucose (2% w/v) [35]. Colonies were scraped from the plates and stored in 2×YT, 1% glucose, 50% glycerol at −80°C. Recombinant phage particles were prepared as previously described [36]. Briefly, the library stock was grown to log phase, infected with M13KO7 helper phage (GE Healthcare), and amplified overnight in 2-YTAK (2YT containing 100 µg/mL ampicillin and 50 µg/mL kanamycin) at 37°C. Phages were further purified and concentrated by polyethylene glycol (PEG) precipitation.

### Selection of VHHs

1×10^12^ plaque-forming units of phage-VHHs prepared from each library were combined for panning against the expressed full length M2 protein coated on Maxisorp immunotubes (Nunc). 10 µg of M2 protein was used in the first round and reduced to 1 µg in the following rounds. Non-specifically absorbed phages were removed by intensive washing with PBS-T (0.1% Tween-20). Bound phages were eluted with 100 mM triethylamine, immediately neutralized with 1 M Tris-HCl (pH 7.4), and subsequently amplified by infecting exponentially growing *E. coli* TG1. The selected phages were amplified with helper phage M13KO7 and purified using polyethylene glycol (MW6000)/NaCl precipitation for further rounds of selection as described [32] . Randomly picked phage-VHH clones were subjected to subtractive binding to native Madin-Darby canine kidney (MDCK) cells (ATCC, CCL-34) and influenza virus infected MDCK cells by ELISA after four rounds of panning. Briefly, MDCK cells (4.5×10^4^/well) were cultured in DMEM containing 10% FBS in 96-well flat bottom plates for approximately 12 h to form confluent cell monolayers and then infected with influenza A virus (MOI = 1) in serum-free DMEM at room temperature for 30 min. The cells were then washed and cultured in DMEM containing 0.5% BSA and 1 µg/ml Tosylsulfonyl phenylalanyl chloromethyl ketone (TPCK)-treated trypsin for 24 h. Uninfected cells were used as a negative control. Cells were blocked with PBS containing 4% nonfat milk and then incubated with phage-VHHs in PBS containing 2% BSA. Specifically bound phages were detected by addition of horseradish-peroxidase-conjugated mouse anti-M13 (GE Healthcare) with the color developed by adding TMB substrate. VHH phage clones with *A*450 value>1.0 were scored as positive; whereas, *A*450<0.2 was considered as negative for binding to virus-infected cells.

### Expression and purification of VHHs

The VHH genes from the selected clones were re-cloned into pET22b (+) vector (Novagen) and transformed into *E. coli* BL21(DE3) (Novagen). Large-scale production of recombinant VHHs was performed in shaker flasks by growing the bacteria in 2×YT supplemented with ampicillin until OD600 reached between 0.6 and 0.9. VHH expression was induced with 1 mM IPTG for 16 h at 28°C. Cells were pelleted, resuspended, and subjected to osmotic shock. The supernatant was loaded onto a Ni-nitrilotriacetic acid (Ni-NTA) superflow Sepharose column (Qiagen), washed, and eluted with 250 mM imidazole. The eluted fractions were concentrated on Millipore concentrators with a molecular mass cut-off of 3 kD and dialyzed in PBS.

### ELISA

10 µg/ml A/Puerto Rico/8/34 (H1N1) virus was passively adsorbed onto 96-well plates in 100 µl PBS/well overnight at 4°C. The virus-coated plates were blocked with PBS containing 4% BSA for 1 h at 37°C. After washing with PBST (1×PBS with 0.1% tween), 50 µl of VHHs and 50 µl blocking agent (1×PBS with 4% BSA) were added to each well and incubated for 1 h. Binding of VHHs was detected with HRP-conjugated rabbit anti-his antibody (Abcam), visualized with TMB substrate (Thermo-Fisher), and quenched with 1 M H_2_SO_4_. The plates were read at 450 nm. ELISA for the expressed full-length M2 protein or the 23mer synthetic peptide of M2e conjugated to KLH was done essentially the same as described above with the concentration of the corresponding protein at 1 µg/ml. The mouse monoclonal anti-M2e antibody, 14C2, was purchased from Santa Cruz Biotechnology (Santa Cruz, California).

### Surface plasmon resonance

The binding kinetics and affinity of the VHH M2-7A and 14C2 antibody for the purified full length M2 protein were measured by surface plasmon resonance (SPR) using a Biacore 3000 instrument (GE Healthcare). Recombinant M2 protein was immobilized on to a CM5 sensor chip in 10 mM sodium acetate buffer, pH 4.0, via amine groups using an Amine Coupling Kit (Pharmacia Biosensors). One channel on the chip was not coated and used as a negative control. Binding kinetics for VHH M2-7A was collected at six concentrations in 2-fold serial dilution down from 1000 nM to 31.25 nM. At the end of each injection, running buffer (10 mM HEPES, 0.3 M NaCl, 3.4 mM EDTA, 0.005% surfactant P20, pH 7.4) was applied for 300 s, followed by the regeneration of CM5 chip using 6 ul of 50 mM NaOH. Binding kinetics were evaluated using a 1∶1 Langmuir binding model with mass transfer control.

### Construction of M2 expressing cell lines

The wild-type M2 gene (M2wt, amantadine-sensitive) of the influenza virus A/Hong Kong/8/68 (H3N2) was synthesized at Takara (Dalian, China). The mutant M2 gene (M2mu, amantadine-resistant) carrying S31N/L26I double substitutions were generated by PCR-directed site-specific mutagenesis (QuikChange®,Stratagene) on M2wt DNA template. The genes were inserted into the pCDNA4/TO plasmid (Invitrogen, Carlsbad, CA, USA) between BamH I and Xba I sites. T-REx-293 cells (Invitrogen) were maintained in Dulbecco's modified Eagle's medium (DMEM) supplemented with 10% fetal calf serum and penicillin-streptomycin (100 U/ml and 100 µg/ml, respectively). T-REx-293 cells were transfected at 80% confluence in a 60 mm dish with 5 µg of plasmid DNA in 10 µl of lipofectamine 2000 (Invitrogen). 24 hours after transfection, cells were passaged at 1∶10 dilution into fresh growth medium containing 200 µg/ml of zeocin (Invitrogen). After 14 days of zeocin selection, tetracycline (Sigma-Aldrich) at a final concentration of 1 µg/ml was added to the cell culture for 24 h to induce M2 expression. The expressed M2 protein was subject to western blotting analysis.

### Flow cytometry

M2wt-T-REx-293 cells were cultured in DMEM containing 10% FBS and induced for M2 expression with tetracycline at 2 µg/ml. Cells were harvested after 24 h and washed with PBS/2.5 mM EDTA. Aliquots of 1×10^6^ cells were resuspended in 0.1 ml PBS containing 0.5% BSA and incubated with 5 µg of M2-7A, 6D or 0.5 µg of 14C2 control antibody at room temperature for 60 min. The uninduced M2wt-T-REx-293 cells were also incubated with antibodies in the same manner. Cells were washed three times with PBS, followed by immunostaining with 1 µl of rabbit anti-His-FITC antibody (Abcam) or goat-anti-mouse IgG-FITC in 0.1 ml PBS containing 0.5% BSA for 30 min. Cells were washed as above and analyzed on a BD FACSCalibur™. For each sample, at least 5000 events were collected and the data analysis was performed with CellQuest program (BD).

### Immunofluorescence staining

MDCK cells (ATCC, CCL-34) were cultured on sterile glass cover slips overnight at 37°C, treated with A/Puerto Rico/8/34 at a multiplicity of infection (MOI) of 1 for 2 h at 37°C. The culture media was then replaced and the infected MDCK cells were cultured for an additional 24 h. Cells were fixed in 4% paraformaldehyde for 20 min, permeabilized with 0.5% Triton X-100 for 10 min at room temperature, and washed with PBS. The cover slips were incubated with the VHH antibodies in PBS+5% BSA solution at room temperature for 1 h. The cells were washed in PBS four times for 5 min each, stained with rabbit anti-His-FITC antibody for 1 h in dark, washed four times with PBS, and counterstained with 1 µg/ml DAPI for 2 min. Photographs were taken on a Leica microscope.

### *In vitro* plaque reduction assay

MDCK cells were cultured on 12-well plates (Costar) and incubated overnight at 37°C with 5% CO_2_ to near-confluence. Equal number of A/Hong Kong/8/68 (amantadine-sensitive) or A/Puerto Rico/8/34 (amantadine-resistance) influenza A virus (approximately 40–60 pfu) was diluted into 0.3 ml DMEM containing 0.5% BSA and incubated with VHH M2-7A of various concentrations for 30 min at 37°C. The plates were washed once with phosphate-buffered saline (GIBCO), pH 7.2, and the virus-VHH mixture was added to each well. Following a 1 h infection at 37°C, the viral inoculum was removed from the cell monolayer. The cells were washed once with phosphate-buffered saline (GIBCO), pH 7.2 and then overlaid with MEM containing 0.5% BSA, 0.8% agarose, 1 mg/ml TPCK-treated trypsin, the corresponding concentration of M2-7A, 6D and amantadine. Plates were incubated at 37°C for 2–3 days, and cells were fixed with 4% paraformaldehyde containing 0.01% Triton X-100 for 1 h. The agar overlay was removed and blocked with 1× PBS containing 5% BSA and 0.05% Tween 20 for 1 h at room temperature. The cells were then incubated overnight with mouse anti-NP monoclonal antibody (Southern biotech). The cell monolayer was washed with PBS, incubated for 1 h at room temperature with HRP linked anti-mouse secondary antibody, then washed three times with PBS and stained with AEC staing KIT (Sigma). The average number of plaques per sample was determined and the percentage for plaque inhibition was calculated by (1-b/a)100%, where b represents plaque number of treatment group, a for the untreated control.

### Cell viability based assay

M2-T-REx-293 cells (1×10^4^) were seeded into 96-well microtiter plates and grown in medium with 1 µg/ml of tetracycline for 24 h. The cells were incubated with M2-7A, amantadine, or control VHH, 6D (in PBS, pH 7.4) for 30 min at 37°C after replacing the medium, then treated with pH 5.8 buffer (50 mM MES, 25 mM HEPES in PBS) containing the above samples for 3 h, followed by recovery in complete medium for 24 h. 10 µl of Cell Counting Kit-8 (CCK-8, Dojindo, Japan) was added to each well for 3 h to measure the *A*450 by UVstar-Microplates Synergy HT.

### VHH efficacy in mice

All animal studies were conducted according to protocols approved by the Institutional Animal Care and Use Committee of Guangzhou Institute of Biomedicine and Health (GIBH), Chinese Academy of Sciences (Animal Welfare Assurance: #A5748-01; IACUC Permit Number: 2010039). Four groups of mice (10 per group, female 6- to 8-week-old BALB/C) were intranasally inoculated with 10×LD50 A/Puerto Rico/8/34 (H1N1). At 24 and 48 h postinfection, the mice were received i.p. injections of a 200 µg/100 µl dose of VHH M2-7A (binding to M2), control VHH 6D (No binding to M2), and the control group received PBS injections only. Mice were weighed daily for 2 wk and euthanized when weight loss exceeded approximately 30% of the preinfection body weight. To determine the lung viral titer, the infected lungs were collected from three animals per group on days 6 after infection, snap frozen in liquid nitrogen and homogenized in cold MEM containing BSA (MEM/BSA). The plaque assay, as described above, was used to measure viral titers from the clarified lung homogenates.

## Results

### Identification of anti-M2 single-domain antibodies (VHHs)

To generate VHHs against M2 ion channel, we constructed synthetic antibody libraries based on a universal VHH framework, cAbBCII10 [34]. Overlapping PCR strategy was employed to orderly link all oligonucleotides (Fig. 1). The library diversity relied on diverse composition of amino acids in the CDR3 regions and was further expanded through combination of sub-libraries (around 10^10^ for each sub-library) of different CDR3 lengths (9–20 amino acids). To select VHHs with specificity toward native M2 tetramer, we used full length M2 protein as a coating antigen. In the presence of detergent (1% OG), purified full length M2 protein formed oligomer complexes (unpublished observation). After four rounds of panning, the selected VHH clones were tested for binding to M2 protein expressed on the surface of MDCK cells infected with influenza A/Hong Kong/8/68 (H3N2). About one hundred clones were obtained that bound to infected but not mock-infected MDCK cells. Six enriched VHHs with strong binding activities (ELISA *A*450>2.0) were expressed in *E. coli* and purified by immobilized metal affinity chromatography. The binding activity and specificity of the VHHs to M2 protein were confirmed by ELISA and the neutralizing activity for influenza A virus was examined by plaque inhibition assay. One of the isolated VHHs, M2-7A, showed more potent virus inhibition activity than others, and was further investigated in this study.

### Binding properties of VHH M2-7A

**Figure 2:**
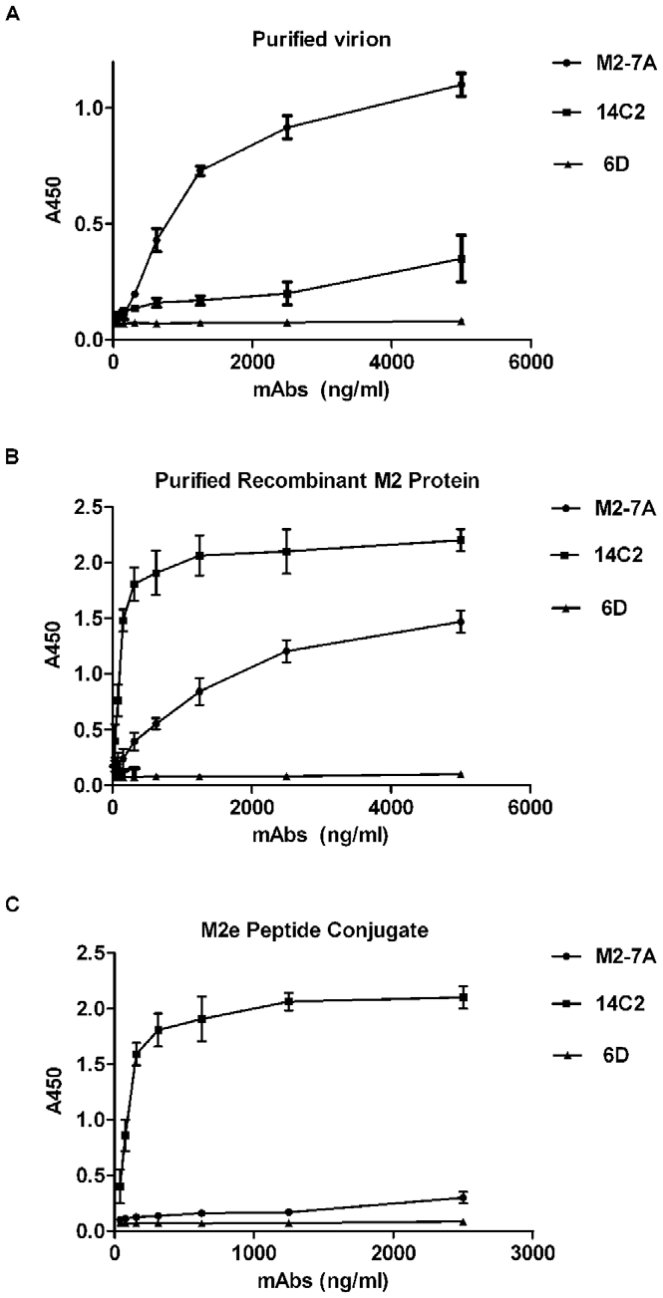
Binding properties of M2-7A. (A) Purified influenza virus (A/Puerto Rico/8/34), 10 µg/ml, (B) Recombinant full length M2 protein, 1 µg/ml, (C) 23-mer synthetic peptide of M2e conjugated to KLH, 1 µg/ml, were coated on ELISA wells and incubated with M2-7A ,14C2 (murine anti-M2 antibody), or 6D (control VHH). The assay was done as described in Methods. Secondary antibodies used: HRP-labeled goat anti-his for M2-7A and 6D; HRP-labeled goat anti-mouse for 14C2.

M2-7A was selected on the basis of its binding to full length M2, influenza-infected MDCK cells, and its ability to inhibit virus replication. To further analyze its binding to M2 protein, M2-7A was expressed in *E. coli* BL21(DE3) and purified to homogeneity. Binding of M2-7A to viral particles (A/Puerto Rico/8/34, H1N1), full length M2 protein, and M2e peptide conjugate were evaluated by ELISA. VHH 6D was used as a negative control, which has the same framework sequence and length as M2-7A but no detectable binding to M2 protein. The M2e-specific murine mAb 14C2 [23] was employed as a positive control. As shown in Fig. 2, M2-7A bound well to both purified A/Puerto/Rico/8/34 (H1N1) virus and the full length M2 protein but weakly to the 23-amino acid M2e peptide conjugate. In contrast, 14C2 bound with similar strengths to both M2e peptide conjugate and recombinant full length M2 protein. Interestingly, 14C2 did not show strong binding to A/Puerto Rico/8/34 virus whose M2e is identical to that of the full length M2 (A/Hong Kong/8/68) and the 23-amino acid M2e conjugate (Fig. 2A). The control VHH 6D, showed little binding to either virion, full length M2, or M2e peptide conjugate. These results indicate that M2-7A recognizes an epitope on the native M2 protein but not on the M2e peptide.

The binding affinity of M2-7A for M2 protein was measured by surface plasmon resonance (SPR) using a Biacore 3000 instrument (GE Healthcare). The recombinant full length M2 protein was directly immobilized on a CM5 sensor chip. M2-7A and14C2 were serially diluted and injected over the chip. To reduce non-specific binding, 0.3 M NaCl was added to the running buffer. The data output represented the value of the observed response units (RU) from the sample cells minus the RU from a reference cell. The association (ka), dissociation (kd) rate constants, and the dissociation constants (Kd = kd/ka) were evaluated using BIA evaluation 3.1 software (GE Healthcare). M2-7A and 14C2 have ka values of 1.1×10^4^ and 2.27×10^5^ M^−1^ s^−1^, kd values of 4.34×10^−4^ and 9.34×10^−5^ s^−1^, respectively. The corresponding Kd value of 14C2 for M2 protein is 4.12×10^−10^ M, comparable to previously published data [20], and the Kd of M2-7A is 3.95×10^−8^ M (Table 1), which is within the range of Kd values most VHHs have for their targeted antigens. This result showed the moderate binding affinity of M2-7A to the full length M2 protein, consistent with previous ELISA data (Fig. 2B). It should be noted that the Kd of M2-7A determined by Biacore may not truly reflect its binding affinity to M2 tetramer, since the immobilized M2 protein sample on the sensor chip also contained M2 monomer, in addition to oligomers including tetramer and octamer (Unpublished observation). Preferential binding of native M2 protein over M2e-KLH conjugate by M2-7A suggested that the actual binding affinity of M2-7A to native M2 tetramer could be stronger than being measured under the current experimental conditions.

**Table 1:** Kinetic rate and dissociation constants of M2-7A and 14C2 to the full length M2 protein.

Ab	ka (M^−1^ s^−1^)	kd (s^−1^)	Kd (M)
M2-7A	1.10×10^4^	4.43×10^−4^	3.95×10^−8^
14C2	2.27×10^5^	9.34×10^−5^	4.12×10^−10^

### Recognition of native M2 on the cell surface by M2-7A

**Figure 3:**
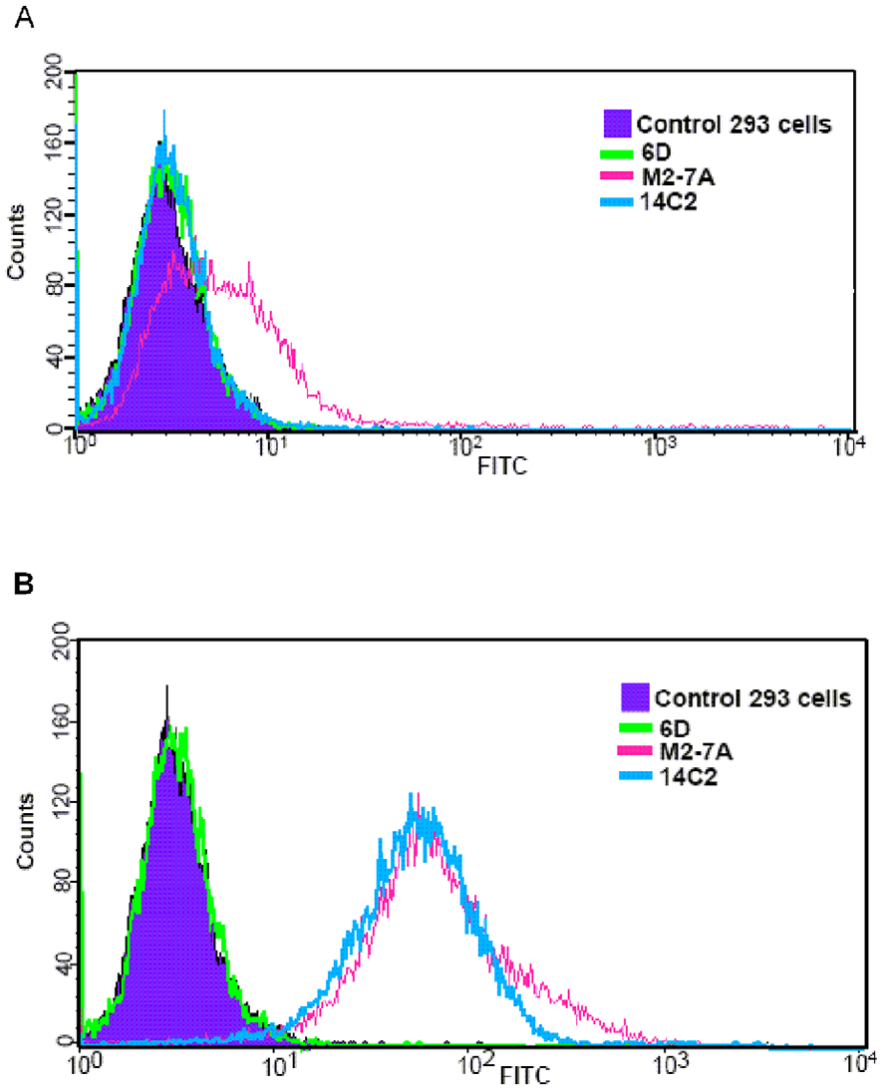
Staining of M2-expressing 293 cells with anti-M2 antibodies. The analysis was performed by flow cytometry with FITC-anti-His tag (M2-7A and 6D) and FITC-anti-mouse IgG (14C2). Cells were non-induced (A) or induced with 2 µg/ml tetracycline for 24 h (B).

**Figure 4:**
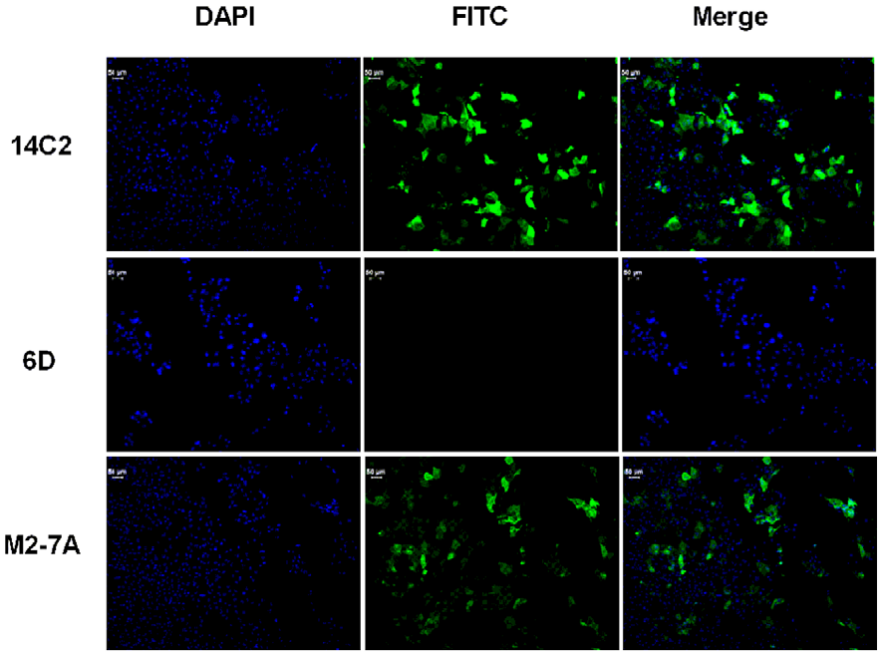
Immunofluorescent staining of influenza infected cells. MDCK cells were infected with A/Puerto Rico/8/34 at an MOI of 1 for 2 h, cultured for 24 h, and fixed with 10% paraformaldehyde. The infected cells were then incubated with control VHH 6D, M2-7A, and 14C2 antibodies then stained with either FITC-rabbit-anti-His IgG (6D and M2-7A) or FITC-goat-anti-mouse IgG (14C2).

The 14C2 mAb showed weak binding to A/Puerto Rico/8/34 viral particles (Fig. 2A) which was in agreement with the previous publication [25]. A small number of M2 molecules on the virion could affect the measurement by ELISA, and another possibility is that the extracellular domain of M2 on the virion is not accessible to 14C2. To address these issues, we next developed a eukaryotic expression system to direct M2 protein onto cell surface where M2 forms a tetrameric ion channel as on the virion. T-REx-293 stable cells expressing M2 upon the induction by tetracycline were established. We then utilized flow cytometry to analyze the binding of M2-7A to M2 protein. As shown in Fig. 3, both 14C2 and M2-7A were able to bind to M2-expressing cells; whereas, control VHH 6D did not show any binding. Furthermore, we investigated the binding of antibodies to M2 on infected cell surface by immunofluorescence staining. Both 14C2 and M2-7A gave strong fluorescence staining on MDCK cells infected with A/Puerto Rico/8/34 virus (Fig. 4). In sharp contrast, control VHH 6D, showed no staining. Taken together, these results demonstrated M2-7A as well as 14C2 bound to native M2 protein on the cell surface.

### *In vitro* viral neutralization activity of M2-7A

**Figure 5:**
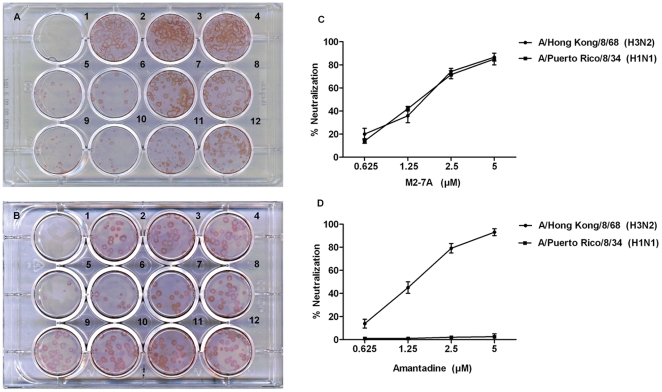
*In vitro* plaque inhibition of viral infection by M2-7A. MDCK cells were infected with A/Hong Kong/8/68 (H3N2, amantadine sensitive) (Plate A), and A/Puerto Rico/8/34 (H1N1, amantadine resistant) (Plate B). In both plate A and B, well 1 was mock-infected; Well 2: virus only; Well 3: virus +PBS; Well 4: virus+6D (5 µM,VHH control); Wells 5–8: virus+M2-7A (5, 2.5, 1.25, 0.625 µM); 9–12: virus+amantadine (5, 2.5, 1.25, 0.625 µM). The percentage of neutralization was determined by (1-b/a)×100%, where b represents plaque number of treatment group, a for the untreated control (well 2), and shown in graph C for M2-7A and D for amantadine, respectively.

The 14C2 mAb has been demonstrated to inhibit influenza virus replication *in vitro* by plaque inhibition assay [23]. However, this antiviral effect occurred only against certain strains such as A/Udorn/72 and A/HK/8/68 but not A/PR/8/34 and A/WSN/33. Likewise, we utilized this assay to determine whether M2-7A possesses viral inhibition activity. Purified M2-7A was incubated with either A/HK/8/68 (amantadine-sensitive) or A/PR/8/34 (amantadine-resistance) viruses and the antibody-virus mixtures were then added to MDCK cells for infection. The overlaid agarose also contained M2-7A during remaining periods of the assay. The control VHH 6D and amantadine were similarly tested. The viral titer and optimal antibody concentration were determined by pilot experiments. As shown in Fig. 5, M2-7A reduced plaque number with similar potency for both amantadine-sensitive and resistant viral strains. The inhibition was dose-dependent with a minimal inhibitory concentration at 1.2 µM of M2-7A (12.5 µg/mL). Control VHH 6D had little effect on plaque inhibition. These results demonstrated that M2-7A was able to inhibit replication of both amantadine-sensitive and resistant influenza strains. The ability to contain amantadine-resistant influenza A virus would give M2-7A an advantage fighting a larger spectrum of influenza infection.

### Protection by M2-7A from lethal viral infection

**Figure 6:**
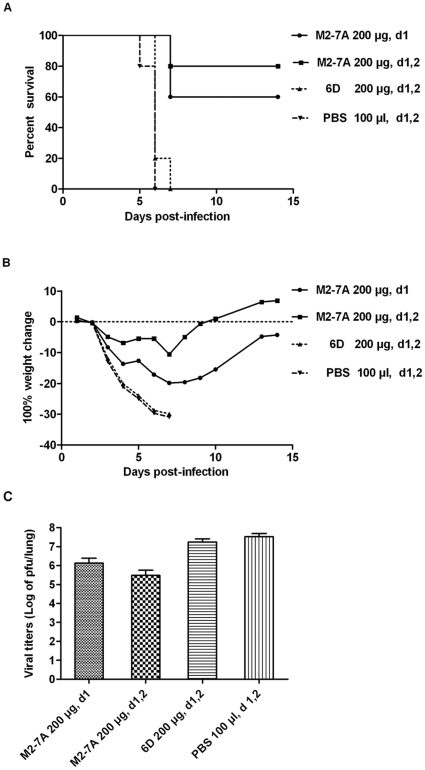
Therapeutic efficacy of M2-7A in mice. Mice (n = 10) were infected by intranasal inoculation with 10×LD50 A/Puerto Rico 8/34 (H1N1), followed by 2 i.p. injections with mAbs at 24 h, 48 h, post-infection and weighed daily for 14 d. (A) Percentage of survival; (B) Percentage of weight change; (C) Lung viral titers were determined from three mice per group at 6d postinfection. 6D serves as VHH control.

We next set out to determine the protective activity of M2-7A in a lethal challenge model of influenza infection in mice. Published studies showed that early treatment within 24 h post-infection and multiple doses were critical for efficacy [20]. Similar therapeutic regimens were applied in our study. Mice were challenged with a lethal dose (10×LD50) of mouse adapted influenza A/Puerto Rico/8/34 and the viability as well as weight change were monitored daily. Mice treated with 200 µg of M2-7A 24 h after viral challenge followed by an additional treatment at 48 h time point were mostly protected from a lethal dose of viral infection (Fig. 6A). In contrast, none of the PBS-treated or the control VHH 6D-treated mice survived the infection. Two-day consecutive treatment showed higher protective efficacy (80% survival) than a single dose treatment (60% survival), suggesting that not only early intervention but multiple treatments as well are effective in subduing the viral infection. The M2-7A-treated mice exhibited weight loss from day 4 to 8 post-infection followed by a gradual weight gain in surviving animals through the end of the study on day 14 (Fig. 6B). The lung viral titers were measured on day 6 after infection. The administration of M2-7A in both regimens substantially reduced viral titers in the lung compared to the control group (Fig. 6C). These results indicate that M2-7A protects mice from influenza A viral infection and may be further developed as a promising therapeutic agent in human.

### Effect of M2-7A on viability of M2-expressing cells

**Figure 7:**
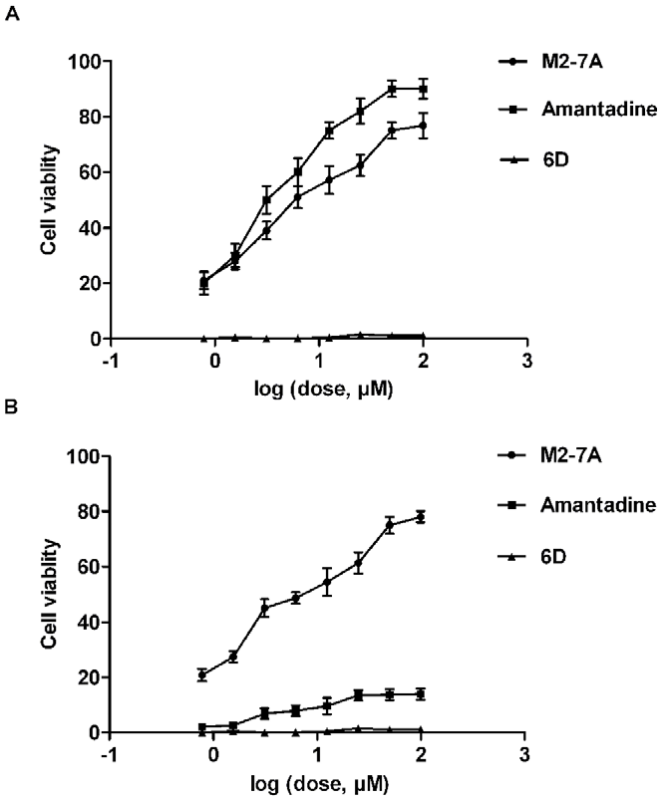
M2-7A protects M2-expressing cells from pH-induced cell mortality. M2wt-T-REx-293 cells (A) and M2mu-T-REx-293 cells (B) were induced by tetracycline for 24 h, incubated with M2-7A, amantadine, or control VHH 6D (in PBS, pH 7.4) for 30 min at 37°C, and then treated with pH 5.8 PBS containing antibodies or amantadine for 3 h. Cells were maintained in complete medium for another 24 h and then assayed using Cell Counting Kit-8 according to the provider's instruction.

As an integral membrane protein of influenza A virus, M2 forms a proton-selective ion channel. Previous studies have shown that low pH can activate the channel and cause M2-expressing cells to die [37], [38]. A cell viability-based assay has been developed to identify drug candidates that antagonize M2 protein [39]. We set out to use this assay to test whether M2-7A could block the function of M2 ion channel and maintain the viability of M2-expressing cells. To this end, both wild-type and mutant (double substitutions S31N/L26I, amantadine-resistant) M2-expressing stable T-REx-293 cells were established. Wild-type and mutant M2 stable cells were induced by tetracycline for 24 hours, treated with PBS at pH = 5.8 for 3 hours, and then cultured for another 24 hours. Cell viability was determined by the use of CCK-8 kit. As expected, amantadine protected M2wt-T-REx-293 cells from death but showed less protection for M2mu-T-REx-293 cells. However, the viabilities of both wild-type and mutant M2-expressing cells were maintained by M2-7A in a dose-dependent manner (Fig. 7). Under the same conditions, control VHH 6D provided no protection for either wild-type or mutant M2-expressing cells from pH-induced cell death. These results indicate that M2-7A interferes with M2 ion channel function, likely by blocking the inflow of protons.

## Discussion

Neutralizing antibodies provide immediate treatment options for influenza pandemic emergency, particularly, for acutely exposed people while more time-consuming developments of vaccines and new drugs are ongoing [21]. Despite numerous epidemics and two major pandemics, the ectodomain of M2 (M2e) protein has shown remarkable conservation; namely, it remains essentially unchanged since the first influenza strains were isolated in 1918 [13]. The M2 protein of influenza A virus has thus become a target for both vaccine and antibody development to achieve broad protection from infection of influenza A variants [15], [16], [40]. However, current influenza vaccines have not achieved a significant anti-M2 humoral response due to low immunogenicity of M2e and fewer number of M2 molecules presented on influenza virus particles [40]–[42]. A multivalent M2e vaccine is in clinical trial which may show great promise [18], [27], [43], but perhaps not be effective for poorly responding populations such as the elderly, very young children and immuno-compromised individuals [5]. Passive immunization with monoclonal antibodies would complement such a vaccine, allowing the treatment of disadvantaged people [21]. Indeed, a number of M2e-specific antibodies have been generated in recent years that showed anti-influenza A virus activities both in prophylactic and therapeutic settings. The mechanisms of action by these antibodies are mostly by ADCC or CDC, targeting infected cells but not directly neutralizing the viruses, which could limit their efficacies in eliminating infections [25], [26].

Single-domain antibody (VHH) fragments are emerging as new versatile reagents for the diagnosis and also the therapy of infectious diseases such as RV-induced diarrhea, HIV, and foot-and-mouth disease [44]–[46]. In comparison to conventional antibodies, one of the unique features of VHH is that it is particularly suitable for binding to the pocket or cleft of targeted antigen owing to its small size and long CDR3 [29]. Therefore, VHHs were chosen to target tetrameric M2 ion channel. Synthetic VHH phage display libraries were constructed using universal framework cAbBCII10, which is expressed well, stable in bacteria, and has the plasticity allowing transfer of donor antigen binding sequences without compromising their binding capabilities [34]. VHH libraries with variable CDR3 length (9 to 20 amino acids) were independently constructed and mixed for panning against recombinant full length M2 protein. VHH phages after four rounds of panning were further selected based on their binding to M2 protein on the cell membrane of influenza infected MDCK cells. The candidate VHHs were then evaluated by plaque inhibition assay. This screening/selection approach was designed to isolate VHHs that bind native M2, the tetramer structure essential for its ion channel activity.

M2-7A, one of the six VHH candidates, showed strong affinity not only for the recombinant full length M2 protein but also the native M2 protein on the virion (Fig. 2A and B). However, it failed to bind a 23-amino acid synthetic M2e peptide (Fig. 2C). Flow cytometry and immunofluorescence staining showed M2-7A also recognized M2 expressed on the cell surface (Fig. 3 and 4). Our results demonstrated that M2-7A specifically recognized native M2. We further observed that M2-7A inhibited replication of both A/Hong Kong/8/68 (H3N2, amantadine-sensitive) and A/PR/8/34 (H1N1, amantadine-resistant) viruses (Fig. 5). The finding is, to our knowledge, the first report for an antibody that is capable of targeting both wild-type and amantadine-resistant influenza A viruses *in vitro*. In a mice challenge model, M2-7A was able to protect mice from a lethal dose of A/PR/8/34 when given 1 or 2 days post-infection. The two-day consecutive M2-7A treatment was more efficacious than a single dose treatment (Fig. 6), in support of findings by others [47], [48]. Different from many conventional anti-M2e antibodies whose antiviral activities *in vivo* are mediated through ADCC or CDC, the protection by M2-7A lack of Fc fragment is likely through blockage of M2 ion channel on the virion and influenza-infected cell surface. The *in vivo* efficacy of M2-7 led to the reasonable prediction that M2-7A-Fc would be more potent than traditional anti-M2 antibodies, owing to the combination of neutralization capability with ADCC or CDC activity.

Other factors such as binding affinity and half-life could also affect *in vivo* efficacy of M2-7A, which has a moderate binding strength (Kd = 39.5 nM) for M2 protein. Further affinity maturation based on M2-7A CDRs can be done as described previously [49]. Alternatively, multivalent M2-7A can be engineered, for example, a peptide linker can be placed between two monomers to generate a bivalent VHH with high affinity or avidity and half-life, similar to an anti-vWF nanobody (camel VHH, Ablynx) on phase II clinical trial.

M2-7A was generated with the framework that has high degree of human antibody sequence homology, thus expected low immunogenicity when used in humans. Due to the small size, M2-7A can be easily humanized to lower or avoid immunogenicity. No B- or T-cell responses have been detected in mice and baboons treated with camel VHHs. In addition, camel VHHs against RANKL and vWF have already successfully passed phase I clinical trial (Ablynx NV), indicating that they were not immunogenic to human.

Influenza A virus M2 protein is a 97-residue single-pass membrane protein with its amino termini towards the outside and carboxyl termini inside the virion. It is a homotetramer in its native state with four transmembrane helices forming a channel for proton conductance. The opening of M2 ion channel is essential for viral uncoating inside the host cell. The detailed mechanism of M2 ion channel opening and closing remains to be resolved, despite a general agreement on its channel pore structure and function [50], [51]. Transmembrane residues, His37 and Trp41, are critical for channel activity serving respectively as a pH sensor and gate. Amantadine, an antiviral drug against influenza A virus, affects the opening of M2 ion channel, and the resistance to amantadine occurs with high frequencies. Although amantadine-resistant mutations are mainly in the transmembrane pore, amantadine binding was surprisingly found at the channel's lipid-exposed outer surface [50]. A possible explanation is that binding by amantadine causes conformational change of distant M2 transmembrane pore, affecting the open and close of the channel, whereas mutations suppress the drug action. Our study has demonstrated that M2-7A protected M2-expressing cells from pH shock-induced cell death, and the protection is equally effective for both M2wt- and M2mu (amantadine-resistant)-expressing cells (Fig. 7), strongly indicating at least a partial blockage of proton influx by M2-7A. Although the exact mechanism of the blockage remains to be investigated, we hypothesized that the binding of the extracellular portion by M2-7A causes a conformational change of the M2 ion channel transmembrane helices, thereby, preventing the opening of the channel for proton influx. The binding epitope of M2-7A might be conformational since our selection strategy against native M2 protein was favorable in generating conformation-dependent VHHs. This notion was supported by the ELISA data showing the weak binding of M2-7A to M2e peptide conjugate. Moreover, long CDR3 of M2-7A could form the structure that fits the pocket or cleft formed by extracellular regions of M2 tetramer. Interestingly, CDR3 of M2-7A has 18 amino acids (IHMRSHGHTKQNRTTY) with multiple histidine residues and showed no homology to other five anti-M2 VHHs. The roles of these residues in binding to and blocking M2 ion channel protein requires further investigation.

In conclusion, this study demonstrated the feasibility of generating a novel class of antiviral drugs using synthetic VHH libraries and utility of function-based screening/selection approach for neutralizing antibodies. VHH M2-7A, showed preferred binding to native M2, and potent neutralizing activities for both wild-type and amantadine-resistant influenza A viruses, likely through direct interference with M2 ion channel function. *In vivo* efficacy of M2-7A warranted its further study as a clinical candidate for broad protection against influenza infection in human.
